# Telehealth cognitive behaviour therapy for the management of sleep disturbance in women with early breast cancer receiving chemotherapy: a feasibility study

**DOI:** 10.1007/s00520-024-08554-8

**Published:** 2024-05-23

**Authors:** Emma-Kate Carson, Haryana M. Dhillon, Janette L. Vardy, Chris Brown, Kelly Ferrao Nunes-Zlotkowski, Stephen Della-Fiorentina, Sarah Khan, Andrew Parsonson, Felicia Roncoloato, Antonia Pearson, Tristan Barnes, Belinda E. Kiely

**Affiliations:** 1 Faculty of Medicine and Health, University of Sydney, Camperdown, NSW Australia; 2 School of Medicine, Western Sydney University, Campbelltown, NSW Australia; 3 Macarthur Cancer Therapy Centre, Campbelltown Hospital, Campbelltown, NSW Australia; 4 Centre for Medical Psychology & Evidence-Based Decision-Making, University of Sydney, Sydney, NSW Australia; 5 Faculty of Science, School of Psychology, Psycho-Oncology Cooperative Research Group, University of Sydney, Sydney, NSW Australia; 6 Concord Cancer Centre, Concord Repatriation General Hospital, Concord, NSW Australia; 7 NHMRC Clinical Trials Centre, University of Sydney, Camperdown, NSW Australia; 8 Southern Highlands Cancer Centre, Southern Highlands Private Hospital, Bowral, NSW Australia; 9 Cancer Services, South Western Sydney Local Health District, Liverpool, NSW Australia; 10 Faculty of Medicine, Health and Human Sciences, Macquarie University, Macquarie Park, NSW Australia; 11 Northern Beaches Hospital, Frenchs Forest, NSW Australia

**Keywords:** Sleep disturbance, Insomnia, Breast cancer, Chemotherapy, Quality of life, Telehealth

## Abstract

**Purpose:**

Sleep quality commonly deteriorates in people receiving chemotherapy for breast cancer (BC). We aimed to determine feasibility and acceptability of telehealth-delivered cognitive behaviour therapy for insomnia (CBT-I) in people with early BC receiving (neo)adjuvant chemotherapy.

**Methods:**

Multi-centre, single arm, phase 2 feasibility trial. People with stage I-III BC received 4 sessions of telehealth CBT-I over 8 weeks, during chemotherapy. Participants completed Pittsburgh Sleep Quality Index (PSQI) and other Patient Reported Outcome Measures (PROMs) at baseline, post-program (week 9) and post-chemotherapy (week 24); and an Acceptability Questionnaire at week 9. Primary endpoint was proportion completing 4 sessions of telehealth CBT-I.

**Results:**

In total, 41 participants were recruited: mean age 51 years (range 31–73). All 4 CBT-I sessions were completed by 35 (85%) participants. Acceptability of the program was high and 71% reported ‘*the program was useful’*. There was no significant difference in the number of poor sleepers (PSQI score ≥ 5) at baseline 29/40 (73%) and week 24 17/25 (68%); or in the mean PSQI score at baseline (7.43, SD 4.06) and week 24 (7.48, SD 4.41). From baseline to week 24, 7/25 (28%) participants had a ≥ 3 point improvement in sleep quality on PSQI, and 5/25 (20%) had a ≥ 3 point deterioration. There was no significant difference in mean PROM scores.

**Conclusion:**

It is feasible to deliver telehealth CBT-I to people with early BC receiving chemotherapy. Contrary to literature predictions, sleep quality did not deteriorate. Telehealth CBT-I has a potential role in preventing and managing sleep disturbance during chemotherapy.

Australian New Zealand Clinical Trials Registry (ANZCTR) registration number: ACTRN12620001379909 and date 22/12/2020.

**Supplementary Information:**

The online version contains supplementary material available at 10.1007/s00520-024-08554-8.

## Introduction

Breast cancer (BC) is the most common cancer in women [1] and up to 70% of patients with BC report sleep disturbance [2]. Sleep disturbance includes difficulty initiating or maintaining sleep, or circadian rhythm sleep–wake disorders, which can negatively affect an individual’s health and quality of life [3]. While sleep disturbance is common in the general population, it can be triggered or exacerbated by a cancer diagnosis and its treatment [4, 5]. The causes of sleep disturbance in cancer populations include: changes in inflammatory cytokines and disruption of circadian rhythm; treatment-induced menopausal symptoms; hospitalisation; anxiety and distress in response to cancer; symptoms such as pain; and medications used to manage treatment side effects such as corticosteroids [5, 6].

People with BC receiving chemotherapy have been found to experience poorer sleep, more fatigue and more depressive symptoms during treatment compared to before starting chemotherapy [7]. Transient disruption of the sleep–wake cycle progressively worsens with repeat chemotherapy cycles [8]. Impacts of sleep disturbance include: daytime fatigue, reduced cognitive performance, mood swings, and psychological distress [9]. Sleep disturbance is a significant public health problem, resulting in impaired daytime functioning, reduced productivity, diminished health-related quality of life (HRQOL) and increased use of health services [5].

Despite the negative impact of chemotherapy on sleep, few trials have assessed sleep interventions during chemotherapy. Cognitive behaviour therapy for insomnia (CBT-I) is generally regarded as a gold standard treatment for sleep disturbance, with effects lasting beyond treatment completion [10]. CBT-I addresses the cognitive elements of thoughts, beliefs, and emotional reactions that exacerbate poor sleep patterns, along with the behavioural components of Sleep Restriction Therapy and Stimulus Control Therapy, to improve sleep outcomes. Sleep Restriction Therapy aims to improve sleep efficiency by limiting the time spent in bed. Stimulus Control Therapy aims to strengthen the bed as a cue for sleep and weaken it as a cue for wakefulness [10].

A systematic review found CBT-I is associated with clinically and statistically significant improvements in subjective sleep outcomes in people with cancer, and may improve mood, fatigue, and quality of life [11]. In a randomised trial of CBT-I versus control in women who had completed primary treatment for BC and reported sleep disturbance, the CBT-I group reported subjective improvement of sleep, lower frequency of medicated nights, lower levels of depression and anxiety, and better quality of life compared to the control group, with benefits continued for up to 12 months of follow-up [12].

Although effective, accessing CBT-I is difficult for many people, due to cost, availability, and a shortage of trained providers. Telehealth is the provision of healthcare remotely using telecommunication technology, including telephone or videoconference. Telehealth CBT-I has been found to improve sleep, and reduce depression and anxiety symptoms in the general population [13], but there are few studies assessing telehealth delivery of CBT-I in the cancer population. One study demonstrated feasibility of telehealth CBT-I in BC survivors who had completed treatment [14], but no studies have assessed telehealth CBT-I in BC patients whilst receiving chemotherapy.

Telehealth is a promising model for remote delivery of CBT-I as it is potentially more cost effective and accessible than face-to-face [15]. The aim of this study was to determine the feasibility and acceptability of a telehealth CBT-I intervention to prevent and manage sleep disturbance in people with early BC receiving (neo)adjuvant chemotherapy.

## Methods

### Study design

This was a multi-centre, single arm, phase 2 feasibility trial of telehealth CBT-I in people with early BC receiving (neo)adjuvant chemotherapy. The study was approved by the Sydney Local Health District – Concord Zone Health Research Ethics Committee for all sites (CH62/6/2020–018), with governance approved at all participating sites.

### Participants

The study population included people aged ≥ 18 years with stage one to three BC, scheduled to receive adjuvant or neo-adjuvant chemotherapy, and willing and able to complete study questionnaires in English. Participants did not need to report sleep disturbance to be eligible. The study excluded people: with an existing diagnosis of a sleep disorder (e.g., obstructive sleep apnoea, narcolepsy, restless legs syndrome or REM sleep disorder); currently using a continuous positive airway pressure (CPAP) machine; currently receiving CBT for any indication; and regularly working more than one overnight shift per fortnight.

### Procedures

Participants were recruited from outpatient cancer clinics at four hospitals in Sydney, Australia over a 12-month period.

Participants completed Patient Reported Outcome Measures (PROMs) (described below) prior to commencing the telehealth CBT-I program (baseline), after completing the program (week 9), and after completing chemotherapy (week 24). Participants also completed an Acceptability Questionnaire at week 9.

Participants completed a sleep diary recording their daily sleep pattern (bed and awake times), use of steroids, use of rescue medications for sleep, and use of alcohol. Participants completed the diary daily for seven days at each of the assessment points (baseline, week 9 and week 24). The questionnaires and diary could be completed on paper or online.

### Intervention

The telehealth CBT-I program consisted of four 45–75 min sessions delivered every two weeks over eight weeks. Participants commenced the first session before their second chemotherapy cycle. The CBT-I program incorporated standard components of sleep education, sleep hygiene, stimulus control, sleep restriction, development and adjustment of the sleep schedule, cognitive restructuring, and relaxation training (Table 1) [10].

**Table 1:** Telehealth CBT-I program session outline

Session	Outline
1	Welcome and introduction Overview of telehealth CBT-I program Sleep education Sleep hygiene part 1 Stimulus control Set goals and provide assignment for the week
2	Review of session 1 and homework Sleep hygiene part 2 Relaxation techniques Sleep restriction Development of new sleep schedule Set goals and provide assignment for the week
3	Review of session 2 and homework Adjust sleep schedule Cognitive restructuring Set goals and provide assignment for the week
4	Review of sessions 1 – 3 and homework Review and adjust sleep schedule Relapse prevention Review follow-up plan and schedule

Participants had the option to participate in the CBT-I sessions by one or a combination of telephone or videoconference (Zoom Video Communication). CBT-I was provided by provisionally registered psychologists who were students in the University of Sydney Master of Clinical Psychology programme under the supervision of a senior psychologist. The psychologists had received prior training in CBT delivery, and for the trial received further in-person training on CBT-I and sleep disturbance in the breast cancer population supported by a treatment manual and individual session information guides.

### Measures

Acceptability of the intervention was measured using an Acceptability Questionnaire adapted from the 16-item Internet Intervention Utility Questionnaire which has good internal reliability [16]. Participants answered questions on whether they found the intervention helpful, easy to use and if it improved their understanding of sleep disturbance. Responses are rated on 5-point Likert scales from 1 (not at all) to 5 (very) [17].

Sleep quality was measured using the Pittsburgh Sleep Quality Index (PSQI), a well-validated 19-item questionnaire, assessing sleep quality over the past month, with a test–retest reliability of 0.85 for the global score [18]. A higher score indicates worse sleep and a global score of 5 or greater indicates a ‘‘poor’’ sleeper with a diagnostic sensitivity of 99% and specificity of 84% as a marker for sleep disturbances in insomnia patients compared with controls [19]. A change of three points or more has been recommended as the minimal clinically important difference for the PSQI; therefore an improvement in sleep quality in an individual is defined as a ≥ 3 point decrease in their PSQI total score from baseline to 9 weeks, and deterioration in sleep quality as a ≥ 3 point increase [20].

HRQOL was measured using the Functional Assessment of Cancer Therapy-Breast (FACT-B), a validated, widely used breast cancer specific quality of life questionnaire. It incorporates the FACT-General (FACT-G) questionnaire which includes 37 items assessing physical, social/family, emotional and functional well-being, combined with items assessing breast cancer symptoms, body image, bothered by hair loss, worry about effect of stress on illness, worry about members of family getting breast cancer and bothered by weight change [21]. The Cronbach's alpha for the FACT-B total score is 0.90, with subscales ranging from 0.63 to 0.86 [21].

Fatigue was measured using the FACT-Fatigue subscale (FACT-F), a 13-item validated measure of fatigue [22]. The FACT-F subscale has strong internal consistency with a Cronbach alpha range of 0.93–0.95 [22]. Symptoms of anxiety and depression were measured using the Hospital Anxiety and Depression Scale (HADS); a validated and widely used 14 item scale to determine the presence and severity of anxiety and depression in both hospital and community settings [23]. The HADS has a Cronbach's alpha of 0.83 for anxiety and 0.82 for depression [24]. The Distress Thermometer is a validated rating scale (0–10) to determine level of distress in cancer patients, with a Cronbach's alpha of 0.80. [25, 26].

Participants completed a study specific diary to record their daily sleep pattern (bedtime and awake time), use of rescue medications (prescription and over the counter) for sleep, and use of alcohol for sleep.

Demographic characteristics collected included age, country of birth, Eastern Cooperative Oncology Group (ECOG) performance status and menopausal status. Clinical characteristics included cancer receptor status, stage, and chemotherapy regimen received.

## Statistical analysis

### Objectives

The primary objective was to determine the feasibility of delivering telehealth CBT-I during (neo)adjuvant chemotherapy. We defined the intervention as feasible and worthy of further study if at least 70% of participants completed all four sessions of CBT-I. Powering the study to reliably show “success” (80% power) if the true rate was 70%, was chosen by the investigators reflecting both (a) on prior studies reporting CBT-I completion rates in people with breast cancer ranging from 59–84% [27–29] and (b) the minimum completion the investigator team felt would be need for the intervention to be viable. Secondary objectives included: *acceptability* (proportion of women finding the intervention useful and easy to use); and *activity* (impact of CBT-I on: proportion of ‘poor sleepers’ [PSQI ≥ 5]; sleep quality; and use of rescue medications for sleep).

Descriptive statistics, such as means, standard deviation (SD) or count/proportions, were used to describe participant characteristics. Outcomes were compared between baseline and week 9 (telehealth CBT-I program completion) and week 24 (chemotherapy completion) using standard statistical methods, such as t-tests or appropriate non-parametric tests for continuous outcomes, and chi-square tests for categorical outcomes. Effect sizes and 95% confidence intervals were calculated where appropriate. All p-values computed are reported and not adjusted for multiple comparisons. Statistical analysis was conducted using SPSS version 28.

The sample size calculation was based on a Simon 2 stage Phase 2 design [30]. A sample of size of 41 participants provided > 80% power to reject the null hypothesis that the rate of completing all four CBT-I sessions was less than 50% (uninteresting rate). This design had a type I error rate of 5% and power of 80% if the true response rate was 70% (interesting rate). We allowed for up to 10% attrition in the design.

## Results

### Baseline characteristics

Between July 2021 and July 2022, 52 participants were screened, and 41 participants were recruited with mean age of 51 years (SD 9.0, range 31–73). Of the 52 who were screened, one was not eligible (diagnosis of obstructive sleep apnoea) and one not interested. Overall, 50 participants were enrolled, nine decided not to proceed with the most common reasons being busy schedule, being overwhelmed or no longer interested.

Most (68%) had hormone receptor positive breast cancer, 46% had stage two disease, chemotherapy was adjuvant for 56% (Table 2). Most of the CBT-I sessions were delivered via telephone (58%), with the remainder by videoconference; 5% commenced before cycle one chemotherapy and 95% before cycle two. Most (73%) met criteria for poor sleep at baseline (PSQI score ≥ 5).

**Table 2:** Participant characteristics (*n* = 41)

	**Number** *N* (%)*
Age, years	
median (range)	52 (31–73)
mean (SD)	51 (9.0)
ECOG performance status	
0	40 (98)
1	1 (2)
Stage of breast cancer at diagnosis	
I	10 (25)
II	19 (46)
III	12 (29)
Cancer receptor status	
Hormone receptor positive, HER2 negative	20 (49)
Hormone receptor positive, HER2 positive	8 (26)
Hormone receptor negative, HER2 positive	2 (5)
Triple negative	11 (27)
Menopausal status at cancer diagnosis	
Pre-menopausal	17 (42)
Peri-menopausal	7 (16)
Post-menopausal	17 (42)
Timing of chemotherapy	
Adjuvant	23 (56)
Neo-adjuvant	18 (44)
Chemotherapy regimen	
Doxorubicin, cyclophosphamide, paclitaxel	23 (56)
Doxorubicin, cyclophosphamide, paclitaxel, carboplatin	4 (10)
Docetaxel, cyclophosphamide	6 (15)
Docetaxel, carboplatin	8 (19)
HER2-targeted therapy	
Trastuzumab	10 (24)
Pertuzumab	4 (10)

^*^unless otherwise specified

## Primary outcome

### Feasibility

All four telehealth CBT-I sessions were completed by 35/41 (85%, 95% CI 72—93) participants during chemotherapy. This rejected the null hypothesis that the feasibility rate was less than 50% (*p* < 0.01).

Assessment completion rates decreased over time, with 98% and 92% completing the questionnaire and sleep diary respectively at baseline, 76% and 76% respectively at week 9, and 61% and 56% respectively at week 24.’

## Secondary outcomes

### Acceptability

**Fig. 1:**
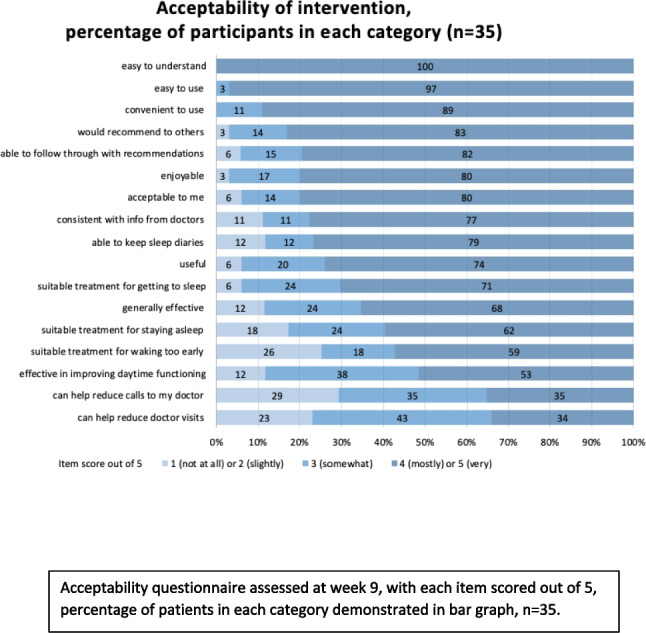
Acceptability of receiving telehealth CBT-I during chemotherapy. Acceptability questionnaire assessed at week 9, with each item scored out of 5, percentage of patients in each category demonstrated in bar graph, *n* = 35

Of 31 participants completing the post-program questionnaire, 100% reported ‘the program was easy to understand’, 89% ‘convenient to use’, 83% ‘would recommend the program to others’, 74% ‘the program was useful’, and 66% believed ‘the program was generally effective’. In total, 70% reported ‘the program as a suitable treatment for getting to sleep’ and 60% ‘the program was a suitable treatment for staying asleep’ (Fig. 1).

### Activity

**Fig. 2:**
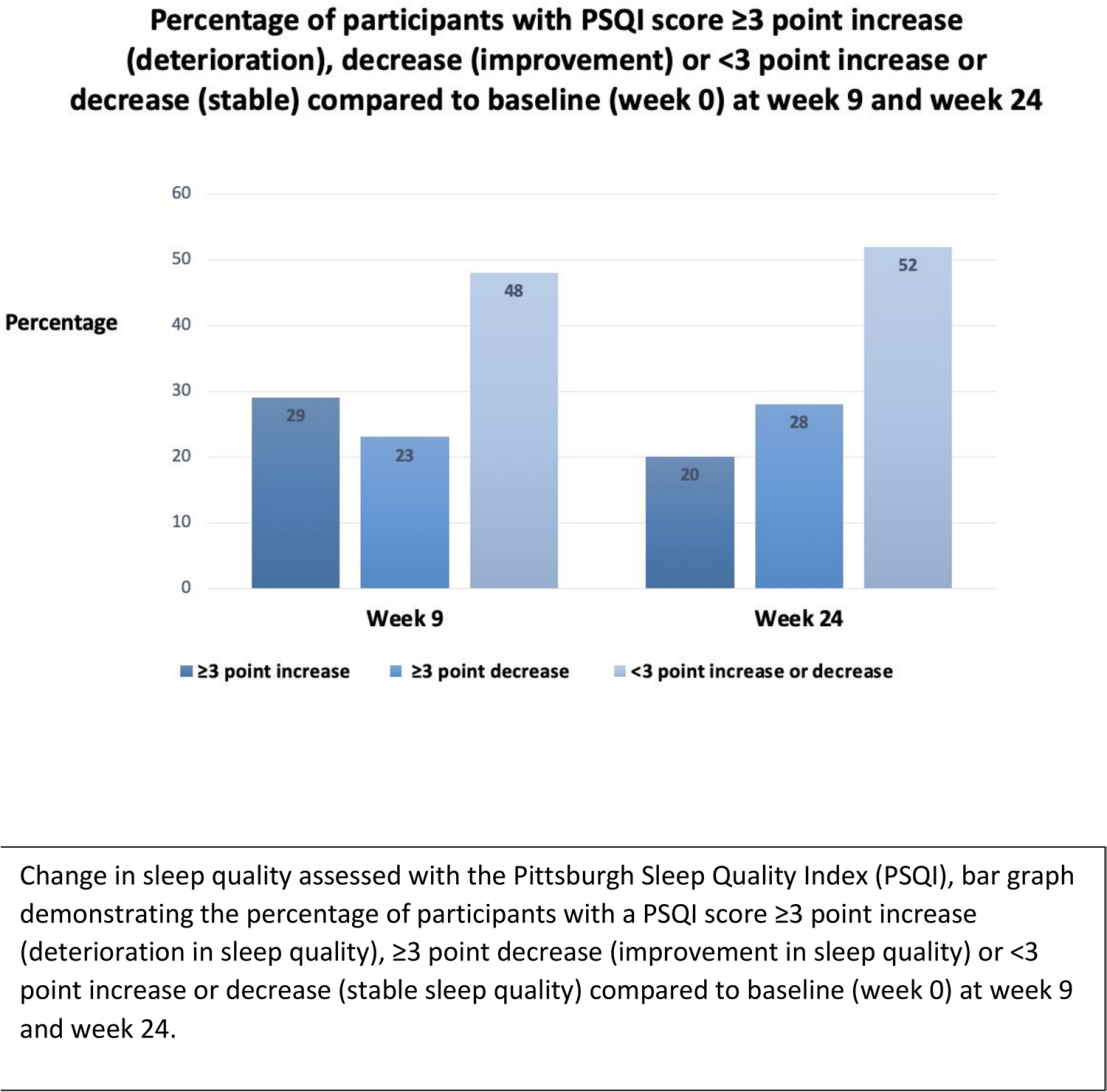
Change in sleep quality (PSQI) from baseline to week 9 and 24. Change in sleep quality assessed with the Pittsburgh Sleep Quality Index (PSQI), bar graph demonstrating the percentage of participants with a PSQI score ≥ 3 point increase (deterioration in sleep quality), ≥ 3 point decrease (improvement in sleep quality) or < 3 point increase or decrease (stable sleep quality) compared to baseline (week 0) at week 9 and week 24

There was no significant difference in the proportion of poor sleepers (PSQI score ≥ 5) at baseline (29/40; 73%) and week 9 (21/31; 68%), (*p* = 0.7) (Table 3). From baseline to week 9, 7/31 (23%) participants had an improvement in sleep quality (≥ 3 point decrease in their PSQI score) and 9/31 (29%) had a deterioration in sleep quality (≥ 3 point increase in PSQI scores) (Fig. 2).

**Table 3:** Sleep disturbance and health related quality of life in participants at baseline, week 9 and week 24

	Baseline (week 0), mean score^*^ (SD, range^†^), *n* = 40	Post CBT program (week 9), mean score^*^ (SD, range^†^), *n* = 31	*p* value, week 0 vs week 9	Post chemotherapy (week 24), mean score^*^ (SD, range^†^), *n* = 25	*p* value, week 0 vs week 24
PSQI	7.4 (4.1, 1–20)	7.4 (4.3, 1–16)	0.97	7.5 (4.4, 2–17)	0.96
PSQI score ≥ 5 N (%)	29 (73)	21 (68)	0.66	17 (68)	0.70
FACT-B	97.6 (17.1, 67–138)	98.1 (22.4, 58–141)	0.92	102.8 (21.7, 50–154)	0.29
FACT-F	31.9 (11.1, 13–51)	28.4 (13.0, 8–52)	0.22	29.5 (12.9, 3–48)	0.44
HADS Anxiety	6.9 (4.3, 1–17)	5.5 (4.3, 0–16)	0.18	6.2 (4.7, 0–17)	0.60
HADS Depression	7.4 (4.1, 0–18)	8.5 (4.3, 0–16)	0.27	6.5 (4.5, 0–17)	0.43
Distress Thermometer	7.4 (4.1, 2–8)	8.5 (4.3, 0–8)	0.27	6.5 (4.5, 0–8)	0.44

^*^unless otherwise specified

^†^Normal ranges: PSQI 0–21, FACT-B 0–148, FACT-F 0–52, HADS Anxiety 0–21, HADS Depression 0–21, DT 0–10

PSQI Pittsburgh Sleep Quality Index

FACT-B Functional Assessment of Cancer Therapy-Breast

FACT-F Functional Assessment of Cancer Therapy-Fatigue subscale

HADS Hospital Anxiety and Depression Scale

The use of recognised medications for sleep (melatonin, doxylamine, and temazepam) was low throughout the study and there was no significant difference in the use of rescue medication between baseline and week 9 or 24 (Table 4).

**Table 4:** Self-reported use of rescue sleep medications* and alcohol at each timepoint

	Baseline (week 0) *n* = 38	Post CBT program (week 9) *n* = 31	*p* value week 0 vs week 9	Post chemotherapy (week 24) *n* = 23	*p* value week 0 vs week 24
Rescue sleep medication*					
N (%) using sleep medication	5 (14)	5 (16)	0.73	3 (13)	0.99
Mean (SD) number of medicated nights/week	0.7 (2)	0.8 (2)	0.80	0.4 (2)	0.28
*Alcohol for sleep*					
N (%) using alcohol for sleep	4 (11)	1 (3)	0.25	3 (13)	0.77
Mean (SD) number of alcohol nights/week	0.2 (1)	0.1 (0)	0.38	0.3 (1)	0.41

^*^includes the following recognised medications for sleep—temazepam, doxylamine, melatonin

### Sleep disturbance at week 24

There was no significant difference in the number of poor sleepers (PSQI ≥ 5) at baseline 29/40 (73%) and week 24 17/25 (68%), (*p* = 0.7); or in the mean PSQI score at baseline, 7.43 (SD 4.01) and week 24, 7.48 (SD 4.31) (*p* = 1.0) (Table 3, Fig. 2).

### Health related quality of life

There was no significant difference in the HRQOL total or domain scores at baseline, week 9 (total score, *p* = 0.9), and week 24, (total score, *p* = 0.3) (Table 3).

### Use of alcohol for sleep

The reported use of alcohol was low throughout the study and there was no significant difference in the use of alcohol for sleep between baseline and week 9 or 24. The proportion of participants using alcohol for sleep was 4/38 (11%) at baseline, 1/31 (3%) at week 9 (*p* = 0.2) and 3/23 (13%) at week 24 (*p* = 0.8) (Table 4).

### Sleep times

The mean sleep duration at baseline was 7:52 hh:mm (range 5:15–10:42), 8:15 (5:54–9:30) at week 9 and 8:06 (6:12–9:32) at week 24.

### Corticosteroids

At baseline 71% of participants self-reported using corticosteroids at least once during the 7 day study diary period, with a mean number of corticosteroids days 1.9 (range 0–6), this decreased to 55% and 1.0 (0–5) respectively at week 9, and 17% and 0.6 (0–6) respectively at week 24.

## Discussion

Our study demonstrated it is feasible and acceptable to deliver telehealth CBT-I to people with early BC receiving chemotherapy, with 85% of participants completing all four sessions. Most participants found the program easy to understand, easy and convenient to use, and would recommend it to others. One quarter of our participants had improvement in their sleep, defined as a PSQI score decrease of ≥ 3.

Several studies have demonstrated receipt of chemotherapy is associated with progressively worse sleep in women with early BC with enduring impairments in sleep-wake activity rhythms measured by actigraphy, as well as significantly poorer (increased) PSQI scores from pre to post chemotherapy [8, 31–33]. Based on these studies, and participants not needing to have sleep disturbance at baseline, we expected to see a deterioration in sleep quality in our study. Instead, we found sleep quality did not deteriorate during chemotherapy with few participants (29%) experiencing a decline in sleep quality, and no significant difference in mean PSQI scores or the proportion with a PSQI score of ≥5 (i.e., poor sleep). We did not expect or see a significant improvement in sleep quality.

A meta-analysis from 2016 demonstrated that cancer survivors treated with CBT-I showed improvements in sleep, which were sustained up to 6 months [34]. Most of the included studies were in BC survivors, after completion of primary treatment. One study determined it was feasible to deliver telehealth CBT-I to BC survivors with sleep disturbance but this study did not include participants currently receiving chemotherapy [14]. Other studies have determined feasibility and efficacy of video or internet-based self-help CBT-I in BC survivors who reported sleep disturbance [27, 28, 35–37], but the interventions were not delivered during chemotherapy. Delivering telehealth CBT-I to patients during chemotherapy may help them develop good sleep behaviours and may prevent sleep disturbance development.

A small number of studies have assessed behavioural interventions for sleep during chemotherapy in people with BC [29, 38–41]. A variety of face-to-face and self-help behavioural interventions were assessed, including individualised sleep plans, brief behavioural therapy, and CBT-I combined with light therapy. Similar to our study, most aimed to prevent and manage sleep disturbance, so participants were not required to have sleep disturbance prior to participation. Subjective sleep assessments either improved or remained stable. While some of these studies have evaluated CBT-I during chemotherapy, to our knowledge, no trial has determined feasibility or activity of telehealth CBT-I in people with early BC whilst receiving chemotherapy.

In our study, we reported the mean PSQI scores, the proportion of poor sleepers (PSQI score of ≥ 5), as well as the proportion with a PSQI score increase or decrease of ≥ 3 (indicating sleep deterioration or improvement, respectively). Other studies assessing sleep in people with BC during chemotherapy, commonly only report mean scores. Some observational studies have reported sleep outcomes using a predefined threshold to define poor sleep (i.e., PSQI) or clinically significant insomnia (i.e., Insomnia Severity Index) [32]. No studies reported the proportion with pre-defined score changes, to indicate sleep deterioration or improvement. We believe reporting these proportions when assessing the impact of cancer treatment or interventions, is more clinically meaningful and relevant to individual patients than mean scores.

In our study, there was no significant difference in HRQOL scores, including the FACT-B global score or for each domain. There also was no significant difference in fatigue, anxiety, depression, and distress. This contrasts with other studies reporting HRQOL, fatigue, anxiety and depression all significantly deteriorate in women with BC during chemotherapy, returning to baseline after completion [7, 31, 33, 42, 43]. The idea CBT-I may prevent deterioration in HRQOL during chemotherapy is worthy of further study. This is supported by trials that have assessed behavioural interventions for sleep in women with BC during chemotherapy and have shown fatigue either improved or remained stable, with no change in depression or anxiety scores [29, 38–40].

Overall, we found the use of both rescue sleep medication and alcohol for sleep were low and did not significantly change throughout the study. This is surprising given the majority (73%) had poor sleep (PSQI ≥ 5) at baseline. This may be due to patient concerns of interactions with chemotherapy. These rates are similar to another study in women with BC receiving adjuvant chemotherapy, which demonstrated 14–23% used at least one sleep medication [44]. They also reported 4% used alcohol, again similar to our population.

## Strengths/limitations

This study is characterised by several strengths, including use of a longitudinal design with assessment of sleep and HRQOL before, during and after chemotherapy, recruitment from multiple sites, and use of the PSQI, a well validated sleep assessment. Our study addresses a common problem, and measured feasibility and acceptability of the intervention. We assessed a non-pharmacological intervention that is simple, convenient, and safe to deliver during chemotherapy without concern of drug interactions or side effects from psychotropic medications. This intervention permits access to a psychologist who does not have to be on site, particularly beneficial to cancer centres with a limited psychology service. The telehealth format is convenient, saving patients time travelling to and from appointments. Telehealth CBT-I may prevent sleep disturbance in this population before it becomes a chronic problem. It has a potential role in preventing and managing sleep disturbance not only in those receiving chemotherapy, but during survivorship and beyond, and further studies are warranted.

Our study has a number of limitations. Firstly, the CBT-I program was condensed into four sessions to aid delivery during chemotherapy. Standard CBT-I generally involves six to eight sessions and we do not know if the shorter duration compromised the intervention activity. Second, we do not have a pre-cycle one chemotherapy baseline assessment on all participants, with 30% of participants completing the baseline assessment pre-cycle one, and 70% pre-cycle two. Given the start of chemotherapy is often an overwhelming time, allowing the baseline assessment to be completed prior to cycle two reduced the burden on participants and improved recruitment. A potential selection bias was that participants with a pre-existing history of sleep disturbance may have been more likely to participate. Similarly, participants more interested in CBT or behavioural interventions may have been more likely to engage with the study. We did not include an objective sleep assessment such as actigraphy. Sleep disturbance may affect cognition, and we did not include a cognition assessment. Our assessment completion rate declined during the study with 61% completing the final week 24 assessment. This was despite participants receiving reminder emails and phone-call from the research team. CBT-I treatment fidelity was not monitored but could be included in a future trial to ensure treatment was delivered in accordance with the treatment manual.

This study provides proof of concept that it is feasible and acceptable to deliver telehealth CBT-I to people with early BC receiving chemotherapy. While the study was not designed to evaluate the efficacy of CBT-I, it is encouraging that we did not see a significant deterioration in sleep quality from baseline to post-chemotherapy. This suggests CBT-I has potential to prevent and manage sleep disturbance in this population. Prevention of sleep disturbance avoids the negative impacts such as fatigue and reduced HRQOL, and the potential need for pharmacological measures. Pharmacological measures provide immediate effects; however, CBT-I produces superior long-term outcomes [45]. Most participants (73%) were poor sleepers at baseline, demonstrating the need for effective, practical, non-pharmacological treatments to manage this common problem.

Future research is needed to assess this intervention in a larger phase 3 randomised study of telehealth CBT-I versus control of standard practice (i.e. sleep hygiene) or waitlist control. In addition to subjective assessments, it would be important to assess sleep objectively using actigraphy, as well as include longer follow-up to assess for long lasting benefits. Trials are also needed to assess this intervention in people with other cancer types treated with chemotherapy.

A stepped care approach comprising a hierarchy of interventions may be the ideal strategy to assess in a future trial, e.g., starting with a self-help intervention and adding expert CBT-I delivered face-to-face or telehealth for those with poorer sleep. This approach may be more cost-effective, and a more appropriate use of scarce resources, addressing the high demand and short supply of CBT-I [46]. This could provide therapy at lower cost, be more convenient, require the lowest treatment intensity and least specialist time. Alternatively, a trial with a matched approach, tailoring the intervention to the precipitating or perpetuating cause of sleep disturbance as well as the severity, using a single or combination of interventions may be more effective but more resource intensive.

## Conclusion

Our results show it is feasible to deliver telehealth CBT-I to women with early BC receiving chemotherapy. Contrary to expectations, mean sleep quality did not deteriorate, with one quarter of participants having improvement in their sleep. Telehealth CBT-I may be a potential intervention for preventing and managing sleep disturbance and maintaining HRQOL during chemotherapy in this population.

### Supplementary Information

Below is the link to the electronic supplementary material.

Supplementary file1 (DOCX 21 KB)

## Data Availability

Data and material are available.
