# Supplementary material for: Telehealth cognitive behaviour therapy for the management of sleep disturbance in women with early breast cancer receiving chemotherapy: a feasibility study
Source: Support Care Cancer. 2024 May 23;32(6):375. doi: 10.1007/s00520-024-08554-8 (PMC11116244; doi:10.1007/s00520-024-08554-8)
Supplement: Supplementary file 1 — Supplementary file1 (DOCX 21 KB) [file 520_2024_8554_MOESM1_ESM.docx]

**Table 1. Completion rates of all four CBT-I sessions and assessments at baseline, week 9 and week 24**

|  | **Assessment completion rate (%)** | | | |
| --- | --- | --- | --- | --- |
|  |  | **Week 0** | **Week 9** | **Week 24** |
| **Telehealth CBT-I four sessions** | 85 | - | - | - |
| **Questionnaire** |  | 98 | 76 | 61 |
| **Sleep diary** |  | 93 | 76 | 56 |

**Table 2. Completion rates of questionnaires at baseline, week 9 and week 24**

|  | **Questionnaire completion rate (%)** | | |
| --- | --- | --- | --- |
|  | **Week 0** | **Week 9** | **Week 24** |
| **PSQI** | 98 | 76 | 61 |
| **FACT-B** | 98 | 76 | 61 |
| **FACT-F** | 98 | 76 | 61 |
| **HADS** | 98 | 76 | 61 |
| **Distress Thermometer** | 98 | 76 | 61 |
| **Acceptability questionnaire** | - | 85 | - |

PSQI Pittsburgh Sleep Quality Index

FACT-B Functional Assessment of Cancer Therapy-Breast

FACT-F Functional Assessment of Cancer Therapy-Fatigue subscale

HADS Hospital Anxiety and Depression Scale

**Table 3. Health related quality of life in participants at each timepoint**

| **FACT-B Domains**^†^ | **Baseline (week 0), mean score (SD, range**[^‡^](https://en.wikipedia.org/wiki/Double_dagger_(typography))**),**  **n=40** | **Post CBT program**  **(week 9), mean score (SD, range**[^‡^](https://en.wikipedia.org/wiki/Double_dagger_(typography))**), n=31** | ***p* value,**  **week 0 vs week 9** | **Post chemotherapy (week 24), Mean score (SD, range**[^‡^](https://en.wikipedia.org/wiki/Double_dagger_(typography))**),**  **n=25** | ***p* value,**  **week 0 vs week 24** |
| --- | --- | --- | --- | --- | --- |
| **Physical well-being** | 18.8 (6.4, 3-27) | 18.1 (5.7, 5-27) | 0.65 | 19.9 (6.4, 4-27) | 0.50 |
| **Social well-being** | 23.0 (4.6, 11-28) | 21.4 (52, 11-28) | 0.20 | 23.0 (5.3, 9-29) | 0.95 |
| **Emotional well-being** | 16.3 (4.8, 7-23) | 17.7 (4.4, 8-24) | 0.23 | 18.3 (4.7, 5-24) | 0.10 |
| **Functional well-being** | 15.6 (5.1, 5-27) | 16.4 (5.2, 8-28) | 0.53 | 17.8 (6.1, 5-28) | 0.12 |
| **Breast cancer subscale** | 24.0 (5.7, 11-36) | 24.5 (8.4, 6-39) | 0.75 | 24.7 (7.2, 8-39) | 0.66 |
| **Global score** | 97.6 (17.1) | 98.1 (22.4, 58-141) | 0.92 | 102.8 (21.7, 50-154) | 0.29 |

^†^ FACT-B Functional Assessment of Cancer Therapy-Breast

[^‡^](https://en.wikipedia.org/wiki/Double_dagger_(typography)) FACT-B domains normal ranges: physical 0-28, social 0-28, emotional 0-24, functional 0-28, breast cancer subscale 0-40, global score 0-148
